# Systematic review on digitals tools used for contact tracing of COVID-19 patients: interim results

**DOI:** 10.1093/eurpub/ckac129.063

**Published:** 2022-10-25

**Authors:** B Unim, I Zile-Velika, J Misins, Z Pavlovska, L Lapão, M Peyroteo, L Palmieri

**Affiliations:** 1 Department of Cardiovascular, Endocrine-Metabolic Diseases and Ageing, National Institute of Health, Rome, Italy; 2 Centre Disease Prevention and Control of Latvia, Riga, Latvia; 3 Population Health, Policies and Services, NOVA University of Lisbon, Lisbon, Portugal

## Abstract

**Background:**

Contact tracing is a public health intervention implemented in synergy with other measures, such as testing, physical distancing, and vaccination, to curb the COVID-19 pandemic. Digital solutions have been developed worldwide to enhance the contact tracing process. The aim of the study was to evaluate the effectiveness and impact of tracking COVID-19 patients using digital tools.

**Methods:**

A systematic literature review was performed on eight online databases to identify observational studies on digital contact tracing, published 2020-2021, in English. Studies identified through the ‘Population Health Information Research Infrastructure’ project were also included. An ad hoc form has been deployed for data extraction of relevant information. Quality assessment of the included studies will be performed using validated tools.

**Results:**

Over 8000 records were identified, of which 27 met the inclusion criteria: 16 modelling and 11 population-based studies. A study was based on GPS technology, four were Bluetooth-based, and others used digital technologies and manual tracing. The uptake rate of the tools ranged 19-100% across the studies. Most studies compared digital contact tracing with other strategies (e.g., no intervention, lockdown). Digital contact tracing was associated with improved identification of contact persons (9 studies), reduction of the effective reproduction number or covid-19 infections (8 studies), and increased effectiveness in combination with other containment measures (9 studies). Security and privacy issues were considered in 8 studies.

**Conclusions:**

Digital contact tracing contributes in reducing further transmission, especially with sufficient population uptake of the applications and in combination with other public health measures. However, its deployment has been limited by security and privacy issues. Further studies are required to investigate the combined impact of digital and conventional contact tracing and enhance privacy and security.

